# Organizational factors behind low sickness absence in Swedish municipalities—An explorative qualitative study

**DOI:** 10.3389/fpubh.2025.1519981

**Published:** 2025-02-21

**Authors:** Åsa Stöllman, Ulrich Stoetzer, Magnus Svartengren, Fredrik Molin

**Affiliations:** ^1^Department of Medical Sciences, Occupational and Environmental Medicine, Uppsala University, Uppsala, Sweden; ^2^Department of Occupational and Environmental Medicine, Uppsala University Hospital, Uppsala, Sweden; ^3^Swedish Work Environment Authority, Solna, Sweden; ^4^IPF, The Institute for Organizational and Leadership Development, Uppsala University, Uppsala, Sweden

**Keywords:** public organizations, organizational factors, organizational health, leadership, employee participation, communication, psychosocial work environment, systematic work environment management

## Abstract

**Introduction:**

This study explores organizational factors that contributes to low sickness absence in Swedish municipalities.

**Methods:**

A qualitative, explorative design was used, including semi-structured individual interviews with 63 managers across 12 municipalities with either high or low sickness absence. Interviews were conducted with Human resources managers, Administration managers and Unit managers in senior care and schools.

**Results:**

The results revealed that municipalities with low sickness absence demonstrate leadership practices that emphasize proximity and accessibility, promote employee development, integrate systematic work environment management, including employee participation into daily routines. Additionally, an open and effective communication was identified as a crucial factor in fostering sustainable organizations. The results show that municipalities with lower sickness absence rates tend to have more sophisticated organizational strategies, especially in clear and manageable areas, it requires strategic planning and well-defined structures.

**Discussion:**

A recurring theme in the analysis is the proximity and interconnectedness observed in leadership, communication, and employee participation, with leaders that show a high degree of involvement in day-to-day activities. This “proximity principle,” emerges as a potential significant factor influencing health outcomes in working life.

## 1 Introduction

Sickness absence is a significant issue for many organizations, not least public organizations, affecting not only the health and wellbeing of employees but also organizational productivity and financial stability. It can have significant negative impacts on both employees and organizations, including decreased productivity, higher healthcare costs, increased personal suffering, and reduced employee morale. Despite this, some public organizations, such as municipalities, have managed to achieve low levels of sickness absence (1, 2).

Sweden comprises 290 municipalities, which bear the primary responsibility for delivering a majority of public services. Certain obligations are mandated by law, encompassing critical areas such as schools, day-care centers, senior care, social services, urban planning, emergency services, and libraries. Municipalities are governed by elected politicians, while day-to-day operations are managed by a variety of employees at different hierarchical levels. Legislation and national guidelines regulate these activities in a uniform way across the country, despite significant variations in geographical size, population structure, and labor force size (3). The sizes of the workforce in the municipalities exhibit substantial diversity, ranging from 299 employees in the smallest municipality to 43,308 employees in the largest (4).

Although sickness absence is mostly related to sickness, its causes are multifactorial, relating to both organizational and individual factors (5–12). MacDonald and colleagues (13) emphasized the importance of organizational factors in studying health outcomes, such as sickness absence. Organizational factors interact in the making of psychosocial working conditions and can also affect health directly, e.g., through decision-making processes. While there are studies aiming at exploring reasons for sickness absence on an organizational level, they are scarce (14–16).

The concept of “organizational health” refers to the overall wellbeing, sustainability, and effectiveness of an organization. There are several perspectives and frameworks that contribute to the understanding of this concept, one of which is the field of organizational psychology and management studies. This perspective often draws on theories of organizational behavior, leadership, culture, and systems theory to explain how different factors contribute to an organization‘s health (17–20).

In today's working life, key factors for success go beyond merely avoiding risks; they also include promoting motivation, cooperation, creativity, and good health (21). To identify factors that have a positive impact, it is not enough to simply reverse known negative factors. Positive factors may be qualitatively different. Most studies have tried to find explanations at the individual level, although sometimes aggregated to the group level. This project, however, focuses on the organizational, strategic level. It considers the factors associated with low sickness absence among employees (22, 23). Positive organizational scholarship (POS) is a subfield of organizational studies that focuses on the positive aspects of organizations, such as positive emotions, strengths, virtues, and flourishing (24–26). It aims to understand how organizations can foster positive experiences, behaviors, and outcomes for their employees, customers, and stakeholders, and how these positive elements can lead to improved performance and wellbeing. POS seeks to provide a balanced view of organizations, moving beyond a narrow focus on problems and negative aspects, and instead highlighting the potential for organizations to be sources of positive impact and growth (27). The intended contribution of this study is to provide scientific insights into the relationship between POS and employee health, including organizational factors that may contribute to lower sickness absence rates.

The term positive deviant cases refers to organizations, individuals, or communities that demonstrate exceptional performance, attitudes, or outcomes in a particular area, despite facing similar challenges or constraints as their peers (28). In other words, these cases are outliers that deviate from the norm in a positive direction. Often studied in the context of organizational or social change, positive deviance highlights examples of organizations or individuals that have found innovative and effective solutions to problems. By examining these outliers, researchers can identify best practices that may be replicated in other settings. Positive deviant cases can provide valuable insights and serve as a source of inspiration and learning for others.

Another theoretical framework to consider is the theory of sustainable healthy organizations, which emphasizes creating and maintaining organizational environments that promote the wellbeing of both employees and the organization as a whole, while also contributing positively to society and the environment (29). The principles of this theory are important factors in creating organizations that prioritize employee health and wellbeing, leading to reduced sickness absence.

Furthermore, the role of human resource management (HRM) practices in achieving low sickness absence is of significant interest (30, 31). Managers in public organizations play a crucial role in promoting the health of employees and reducing sickness absence within their organization (32). However, little is known about how managers in municipalities with low levels of sickness absence integrate health promotion activities into their daily work, so that they become part of the organizational structure and permeate the organization as a whole. By focusing on positive deviant cases (municipalities with low levels of sickness absence), the study identifies specific strategies and practices on an organizational level, used by several managers within the same organization. These practices contribute to their success in promoting employee health and reducing sickness absence (33). Additionally, it provides insights into the role of leadership, communication, and collaboration in promoting employee health.

A previous study in the Swedish private sector identified key health factors in organizations with low levels of sickness absence. The most prominent factors included clear leadership structures, competence, communication, participation, knowledge, and health status procedures. The organizations classified as healthy were also organized in a fair and considerate manner (34, 35).

Thus, the aim of this study was to explore the organizational level factors that characterize municipalities with low sickness absence. In the study, these organizations are referred to as “healthy organizations.”

## 2 Methods and materials

### 2.1 Setting/study context

This qualitative study is part of a larger project focusing on organizational level factors that characterize Swedish municipalities with low sickness absence. An initial register study within the same project revealed no major differences between public organizations concerning quality indicators, such as the number of employees in senior care or in schools, or the operational costs of these services (36). This meant that the conditions for the participating managers did not differ significantly, either in terms of budget or in terms of the number of subordinates.

### 2.2 Design

To achieve the aim of this study, an explorative design was used, with semi-structured individual interviews for data collection. Before the interviews got started, two municipalities served as pilots to test the interview guide. After the data analysis was conducted, the preliminary results were presented to the participants, who were invited to a 3-h meeting for discussion, allowing them an opportunity to share their opinions and reactions. Additionally, a senior researcher not involved in the project read all the interviews to ensure the quality of the conclusions.

### 2.3 Selection of the study group

To identify a selection of healthy organizations, a selection was made based on long-term sick leave statistics. A “healthy organization” is defined as an organization with low levels of sick leave. The selection of municipalities was based on sickness-related absence registers from 2005 to 2007. For the database used in this study, an insurance company provided data on sickness absence over 90 days among employees in municipalities. Since the main focus of the study was to identify healthy organizations, this criterion was prioritized during the selection process.

### 2.4 Sampling of organizations

To ensure representativeness, the selection of organizations included average-sized municipalities (Table 1). Another criterion was to include both healthy and less healthy municipalities from different regions across Sweden, to avoid regional differences that may affect the results. There are big geographical differences in Sweden, with, for example, higher sickness absence in the northern areas. Moreover, municipalities with the highest levels of sick leave were excluded, since these organizations may have specific problems, and any differences that emerge could be attributable to factors that are unique to municipalities with high sickness absence. The focus of this study was to identify factors that distinguish healthy organizations. Finally, municipalities where sickness absence had changed dramatically between 2005 and 2007 were excluded. The reason for this was that other factors may explain the difference. It requires stability over time to avoid other factors that are extraordinary in explaining the difference.

(1) In the first step, a sample of 213 municipalities with between 500 and 2,999 employees was selected from a total of 290 municipalities in Sweden.
(2) In the next step, 10 percent of the municipalities with the highest sickness absence rates were discarded.
(3) From the remaining 193 municipalities, the 35 with the highest and the 35 with the lowest sickness absence were identified.
(4) From the 70 municipalities selected, those where sickness absence had changed dramatically between 2005 and 2007 were excluded.
(5) Thereafter, 15 municipalities with low sickness absence and 15 with high sickness absence were selected to ensure geographical representation across Sweden.
(6) From these remaining 30 municipalities, five with low sickness absence and five with high absence were selected. This was done by using paired selection (1 high sickness absence−1 low sickness absence), to achieve geographical balance, adding them to the selection group as they accepted to participate in the study.
(7) To broaden the investigation, two metropolitan municipalities (in large Swedish cities) were included, each with one district council with low sickness absence and one with high sickness absence.

**Table 1:** Description of participating municipalities.

**Municipality**	Geographical location^**^	Employees^***^(N)	Sickness absence^****^(%)	**Sickness absence per 1,000 employees (N)**
A	N	1,816	8.5	45.5
B	M	2,717	8.2	42.3
C	M	1,243	8.3	41.0
D^*^	M	1,091	7.2	20.8
E	M	1,593	N.A.	41.5
F Metropolitan	S	20,725	8.5	35.0
G^*^	S	991	N.A.	29.9
H	S	1,129	9.0	43.3
I^*^	S	811	6.0	21.4
J^*^	S	886	5.1	23.8
K Metropolitan	M	44,857	9.5	25.4
L^*^	M	1,474	N.A.	19.2

Data from 2006.

^*^Low sickness absence.

^**^North, middle, or southern Sweden.

^***^Data from 2007.

^****^Prevalence of sickness absence over 90 days among employees.

### 2.5 Participants

The data included a total of 63 semi-structured individual interviews with managers from the 12 selected public organizations (Table 2).

**Table 2:** Number of interviews.

**Municipality**	**Human resources managers**	**Administration managers**	**Unit managers**	**Other managers**	**Total number of interviews**
Municipalities, 500–2,900 employees	10	20	19	2	51
Metropolitan municipalities in municipalities >20,000 employees	4	4	4		12
Total	14	24	23	2	63

In 10 municipalities, interviews were conducted with Human Resources managers and Administration managers, as well as Unit managers with a maximum of fifty subordinates in senior care and schools, respectively, the two largest units within the municipalities. In one municipality, two additional managers at a different level were interviewed to obtain additional information. In one municipality, a Unit manager canceled and a replacement could not be found. In the two metropolitan municipalities, interviews were conducted in two districts within the municipality with Human Resources Managers as well as the Directors and Heads of senior care. However, interviews with school personnel were not included in these metropolitan districts, since school-related issues are handled centrally rather than at the district level.

Regardless of the level of sickness absence in the municipality, the unit managers in senior care had between 36 and 40 subordinates, and unit managers in school between 22 and 26 subordinates (data from 2006/2007).

The participants were informed that they could withdraw from the study at any time for any reason and that all material collected would be kept confidential. They accepted participation by telephone and received information about their rights to cancel their participation by email.

### 2.6 Data collection

The interviews for this study were conducted on-site between April 2010 and June 2011, with data collection occurring at or near the participants' workplaces by trained interviewers. Although the data was collected several years ago, it is only now being presented in a scientific context. The study has received notable attention in Sweden across various sectors, including trade unions, the health sector, occupational health services, and the Swedish Agency for Work Environment Expertise (22). The findings have been utilized to create a digital tool for improving the psychosocial work environment, contribute to a Chief Executive Program in municipalities, and disseminated in popular science (23, 37). Unfortunately, the study was not published concurrently in a scientific journal; instead, it is now being reported retrospectively, despite the time gap, because the findings remain relevant and applicable.

One interviewer led the interview, and the other had a more observational role to ensure that all subjects were covered. Each interview lasted ~1.5 h. All interviews were digitally recorded, transcribed verbatim, and then analyzed. The semi-structured interviews were performed by the author (ÅS) and three other researchers in the project: two psychologists (US), one social scientist, and one physiotherapist/behavioral scientist. The interviewers had a broad knowledge of research on the relationship between work and health, and experience in qualitative methods. The interviewer group had regular meetings to discuss interview techniques and the project's methodological approach. Before the interviews started, the interview team was informed about the municipalities' organizational structure to better understand the context. This information was given during a meeting with The Swedish Association of Local Authorities and Regions (SALAR).

An interview guide was developed using the results from a previous similar study conducted in the private sector by the same researchers and covered important insights from earlier research on work environments and organizations (35). The data collection process was preceded by piloting the interview guide in two municipalities, followed by revisions based on feedback.

The guide included open-ended questions to ensure that all relevant topics were covered during the interviews. Additionally, several follow-up questions were asked to gain a deep understanding and description of the situations and narratives presented by the participants, and to make sure that they were understood in the right way. An important aim of the study was to capture and describe the structures and elements within the organization, as opposed to focusing only on personal experiences and impressions. This method is characterized by the interviewer asking the interviewee to provide specific examples of procedures (38).

Areas covered by the interview were:

- Management strategies
- Personnel policy
- Market analysis
- Strategies for organizational change
- Communication
- Employee Participation/Influence
- Work organization and work tasks and resources
- Work Environment

In addition to the interviews, written materials such as occupational health and safety and rehabilitation policies were also taken into account.

### 2.7 Data analysis

A checklist (COREQ) of items that should be included in reports of qualitative research was used as a guide (39).

The transcripts of the semi-structured interviews were analyzed using Qualitative content analysis [QCA; (40, 41)], with a directed approach starting with earlier research findings as guidance for the initial analysis (42). A starting point for both the content and methodology of this study was the experiences gathered from the first part of the research program, performed in private companies (35).

The interviews were analyzed by the four researchers who also performed the interviews, using the software program NVIVO 9 (43) to help categorize the manifest contents of the interviews.

Since the purpose of the study was to explore health factors at an organizational level, it was decided early on to focus on statements that clearly described the strategies of the organizations. The data were initially handled without knowledge of the level of sickness absence, and the first part of the analysis was conducted on material from each of the 10 municipalities.

The analysis was conducted in several steps. In the initial phase, the researchers analyzed the selected interviews individually. Each interview was read through to identify quotes that, on a manifest level, reflected a systematic approach or an established value-system. The contents of the selected quotes were summarized in short sentences, referred to as meaning units. In the next step, the researchers cooperated in pairs, and the same interviews were read through by another researcher. The analysis from both researchers was compared and thoroughly discussed in order to get an overall grasp of the content of the material and to identify similar meaning units. The material was then divided into condensed meaning units, which were merged into categories/themes. These categories/themes captured something important about the data in relation to a specific research question. Once categorization was done, they were compared and discussed within the research group to achieve a consensus on the analysis of the municipalities. The categories/themes from municipalities with low levels of sickness absence were compared with those from municipalities with high levels of sickness absence. The next step involved a deeper analysis of the themes that showed the greatest differences between municipalities with low and higher absenteeism, both in terms of the number of individuals quoted and the total number of citations. Furthermore, these themes were tested in the two metropolitan municipalities. The tests showed no differences regarding the themes between the five municipalities and the two metropolitan municipalities.

The analysis was concluded when saturation was reached, i.e., no new unique or contradictory information was found. An external, skilled researcher, not involved in the project, read through all the interviews in order to find factors that potentially could have been missed in the structured analysis model. At the end of the analysis, two workshops were conducted with municipal representatives in order to get feedback and discuss the findings. The themes that remained after all analysis steps are presented in the results section and will be referred to as health factors at the organizational level.

This is a qualitative study comprising individual semi-structured interviews with managers. Ethical approval was not required from the Regional Ethical Review Board, since the research did not collect or analyze personal data or sensitive information about individuals. The study focused only on the organizational level and did not involve direct interactions with individuals or the handling of their personal data.

## 3 Results

The analysis of the interviews identified four themes: proximity in leadership, learning and development, communication, and systematic work environment management and participation. These themes that characterize municipalities with low sickness absence are more clearly and systematically described, compared with the municipalities with higher sickness absence. There is no ranking of the strength of the results or their importance for health.

### 3.1 Proximity in leadership

Leadership was identified as an important practice in municipalities with low levels of sickness absence. Municipal managers described how they aspired to be in direct contact with as many subordinates as possible, often through unplanned and informal meetings. The managers actively sought information regarding organizational effectiveness through direct contact with lower levels in the organization. For example, this could be done through workplace meetings or by impromptu meetings in the corridor. In some municipalities, the managers' offices are sometimes located close to the lower-level managers in the organization to facilitate communication.

“We are close./…/when I have the time and opportunity, I'm out in the field talking with people. I prefer spontaneous conversations, rather than a scheduled meeting.”

“We are always three or four managers in services, and one functions as site manager. It's ‘the chief of the day'; it is the one who knows how the business flows.”

This direct contact between managers and employees was described by several managers at different levels within the municipalities. It helps managers become familiar with daily operations and handle problems as they arise. Through personalized contact, managers can also follow up on the implementation of decisions. Another manager points to the importance of being visible and accessible:

“I drink a lot of coffee. I like small talk in the hallway, showing myself, seeing people, taking time.”

### 3.2 Learning and development of employees

General training is often conducted based on needs arising from activities associated with changes in laws and regulations, reorganizations, new systems, etc. In some organizations, individual needs for training and development are identified through annual performance reviews. Various levels of management describe opportunities for staff to have their individual wishes met in conjunction with staff training and development, in addition to addressing the immediate and direct needs of the organization.

“I allocate money for [training and development] in my budget. It's important that development is strategic, based on both personal wishes and on goals that [the organization] must achieve.”

Employee training and development were seen as key factors in promoting learning, motivation, and engagement among employees in the municipalities. The municipalities strived to offer a wide range of opportunities for employees to improve their skills and knowledge, and funding for this was allocated within the budget. Another type of learning described by the managers was providing opportunities and encouraging job rotation. The managers stated they encourage employees to try new tasks, acquire new skills, or move to other jobs within the municipality. In some cases, they regard an employee's departure from the municipality for further studies as an inspiration for the rest of the staff.

“We encourage [job rotation]./…/sometimes [an employee] comes to me and wants to work six months somewhere else.”

Job rotation comes more naturally to the larger municipalities**:**

“There is a lot of rotation, and I think that's great. The municipality is large and has many possibilities.”

### 3.3 Communication

Effective communication and collaboration within a positive communication climate were identified as important factors by managers in organizations with low levels of sickness absence. Municipal managers described how they strived to communicate clearly and openly with their subordinates and employees. The municipalities implemented systems and strategies for feedback and the communication of criticism, establishing explicit structures for voicing concerns.

“I try to encourage [employees] to dare to say things about me [as a leader] because I think they influence me as a manager by saying both negative and positive [things].

There are also possibilities for employees to forward criticism and ideas to higher levels in the organization.

“- I: Is it OK to go to you directly?

- A: Yes, there's a reason when they say ‘I have raised this with my boss on several occasions and nothing happens' or ‘we're not talking the same language' or ‘the boss treats me differently' or whatever… It is assured that I will act directly…”

### 3.4 Systematic work environment management and participation

The municipalities with low levels of sickness absence work in a structured and strategic manner, with systematic work environment improvements as part of daily operations. An important part of this is involving employees in the analysis and discussion of safety issues, assigning them different responsibilities. Additionally, making work environment responsibilities visible throughout the organization is described as important.

Clear routines, incorporating employee dialogue, collaboration groups, and documentation are important. In addition to annual audits, it is important to monitor risk assessments regularly and follow up on corrective measures. Managers expressed a desire to work collaboratively with employees to develop and implement these activities, which they believe fosters a sense of shared ownership and engagement among employees.

The municipalities have made it easier for middle-level and first line managers to work systematically with work environment management by using standardized forms and digitalized systems for documentation and follow-up:

“Then, we don't have to run around searching, acquiring, and fixing in the moment; rather, it's about creating a good work environment by thinking about it all the time.”

“So it feels like now, at last, we have it in place, and it's like a toolbox. If there is a work-related incident, then you'll know as a boss exactly what to do.”

The municipalities were found to have high levels of employee involvement, with employees participating in decision-making and problem-solving processes. This was deemed to contribute to employee motivation, engagement, and job satisfaction.

Well thought-out and developed strategies to promote participation were identified as another important factor by managers in organizations with low levels of sickness absence. Municipal managers described how the organization consciously promoted employee participation and encouraged employees to take part in improvement work.

“When I'm out, I try to connect with each one … and ask ‘how do you feel about these changes?'

The employees also had opportunities to escalate concerns and share ideas with those higher up in the organization. Managers described how they appreciated the possibility that employees share their ideas and actively created forums for employees to so.

### 3.5 Summary of findings

The results indicate that managers in municipalities with low levels of sickness absence actively work to foster a culture of health within the organization, prioritizing the integration of systematic work environment management into their daily routines. The results also show that managers in organizations with low levels of sickness absence utilize leadership, effective communication, participation, collaboration between managers and employees, and a deep understanding of the unique context and culture of public organizations.

The study provides an insight into the activities of public organizations and shows that the work of running a complex organization in many respects is carried out by competent and committed people, many with extensive experience.

There are sometimes significant differences between different activities within the same municipality, but the exchange of experience is often limited. As a result, good and creative solutions are not always shared across the organization.

## 4 Discussion

This study aims to explore organizational factors that distinguish Swedish municipalities with low sickness absence, denoted as healthy and sustainable organizations. It exemplifies an approach to examining positive, health-promoting elements at the organizational level. Specifically, the study seeks to ascertain whether the factors crucial for fostering a healthy environment in the public sector align with the health-promoting elements identified and examined in a prior Swedish study conducted in the private sector (35).

Several health promoting organizational factors were identified, which were basically the same main factors as identified in the previous study in the private sector. They were related to management, skills development, communication, employee participation, and sickness absence and health procedures. An additional organizational health factor was found among the municipalities with low sickness rates, indicating that they have managed to integrate health and safety management into their daily routines and have a good knowledge of how systematic work environment management is maintained. The results primarily highlight contrasts and seldom involve clear-cut distinctions between municipalities with low vs. high sickness absence; instead, they present more of a nuanced spectrum.

The key health promoting factors at the organizational level that characterize a healthy public organization are as follows:

- Proximity in leadership
- Learning and development of employees
- Communication
- Systematic work environment management and participation

The objectives and tasks of public organizations in sectors such as healthcare, senior care, and education are largely governed by laws and regulations; therefore, the differences between the organizations in public service seems to be smaller than those in the private sector. A key difference is that municipalities are politically controlled, whereas private sector organizations can more easily organize to be profitable. Public services, on the other hand, are more unpredictable. In municipalities, trade unions also have more influence and power, which allows them to contribute to change and influence the work environment to a greater extent (3).

This inquiry holds particular interest due to the divergent nature of organizational structures and conditions between the public and private sectors. For managers to be able to manage stress within their organizations, they must have a reasonable workload. A study of local public governments found that first-line managers, particularly female managers, often experience high levels of stress and workload, increasing their risk of stress-related mental illness (44). The organizational context that surrounds a leader has an impact on leadership development. Research has shown that the risk of stress and mental illness among managers can be reduced through feedback, encouragement from senior management and supportive standards and principles (45). To prevent the emergence of destructive leadership, organizations should strive to clarify leaders' roles, ensure that leaders' workloads are reasonable, and strive to reduce leaders' stress, as well as assess leaders' personalities during recruitment (46).

The complexities of municipal organizational structures further compound this investigation, given the considerable variations in organizational designs across different municipalities. Additionally, significant distinctions exist in work environments and organizational conditions, with the public sector often characterized by a higher prevalence of female-dominated workplaces and elevated sickness rates. Notably, in 2023, sickness rates stand at 5.9% in municipalities and 3.7% in the private sector (47).

The study drew significant inspiration from the field of Positive Organizational Scholarship (POS), an area of research that delves into the positive processes and attributes of successful organizations and their members. POS places emphasis on understanding how organizations can achieve success, envisioning employees and the organization as exemplifying characteristics such as wellbeing, appreciation, effective collaboration, meaningfulness, resilience, trustworthiness, positive social relations, loyalty, and positive energy, among other factors (24–26). The organizational factors that the current study shows as important for employee health are closely related to the characteristics of positive organizational scholarship. These factors contribute to higher productivity and help strengthen an organization's resilience.

Trust is a key element of the concept “Relational Justice” (organizational justice). This concept incorporates whether employees perceive the organization as fair and benevolent, and depicts how relationships between employees and managers are perceived, where managers are seen as representatives of the organization. Strong relationships between employees and managers increase trust and loyalty, which, in turn, should affect productivity. A range of health outcomes have also been found related to this concept. The theory behind these effects suggests that good relationships and trust within an organization help reduce stress (48–55). It is reasonable to assume that leadership characterized by close involvement with employees' daily operations increases the likelihood of creating good and fair relationships, but also being perceived as an organization that “cares” (34, 35).

Health-promoting leadership stands out in the result as a crucial factor for creating a sustainable organization. A compelling insight is that a key approach to leading health promotion is to be readily available and accessible when employees require support. This entails managers to possess strong social skills and an ability to discern the dynamics within the workplace, as well as understanding what assistance employees might require. In doing so, managers stay informed, not only about day-to-day operations but also about potential signs that someone may be on the verge of falling ill or experiencing other issues that could impact their job performance. Such leadership fosters trust, commitment, and wellbeing among employees, which can contribute to lower rates of sick leave (56–58).

The findings align with the classic Demand-Control-Support model and are corroborated by contemporary research indicating that managerial support is paramount in the workplace. Cultivating a positive relationship with one's supervisor not only promotes employee health, but it is also correlated with heightened motivation among employees. Support from one's superior is a well-known modulating factor in the demand and control model and a lack of support means increased vulnerability to the imbalance between high demands and low control. Supportive leadership has an impact on the perception of wellbeing and quality of life, as well as job satisfaction. It can also be assumed that a present and engaged leader also influences and clarifies tasks and roles, creating fewer conflicts in the workplace (59–64).

The leadership behaviors found in healthy organizations align with the principles of transformational leadership, which emphasizes inspiring employees, articulating a clear vision, and demonstrating consideration of their needs. Past research has established the significance of transformational leadership in fostering job satisfaction and overall wellbeing (65–67).

Another health-promoting factor that emerges is the importance of an open communication climate. Effective communication between colleagues, managers, and across different units/levels is required for well-organized work. Strategies for feedback within healthier organizations enhance employees' capacity to give and receive feedback, facilitating the expression of opinions, ideas, and constructive criticism on work-related matters. The importance of workplace relationships for occupational health is underscored, as effective communication is recognized as a vital component. Robust social support and positive working relationships not only contribute to individual wellbeing but also foster creativity and enhance the assimilation of new knowledge and information.

The findings of this study suggest that healthier organizations cultivate an open communication climate, characterized by clear channels and receptivity to both formal and informal feedback. These organizations actively encourage employee participation, which promotes the emergence of innovative ideas and constructive criticism. This proactive approach raises management awareness and allows for complaints to be addressed more quickly. As a result, employee engagement increases, leading to a greater sense of commitment and satisfaction. A climate of trust also facilitates the smooth implementation of organizational changes (34, 35).

The results of the present study highlight the importance of creating a positive psychosocial work environment within the organization. This needs to do be done in a systematic and persistent manner, as part of a continuous process that incorporates recurring activities into systematic work environment management (68, 69). This aspect emerged as a new health factor compared to the previous study conducted in the private sector. The healthy municipalities had a good knowledge of work environment management and had managed to integrate it into their daily routines. This may be due to the fact that the issue of work environment is more often raised in trade union organizations within municipalities. Healthcare and sick leave procedures are also highlighted as important parts of the work environment management.

The results show that different themes are clearly linked to each other; for example, participation and communication go hand in hand with closeness in leadership. For employers who want to improve their psychosocial work environment, the most important thing is to start by implementing one of these health factors, as this will facilitate positive effects on other factors as well. A common thread in the results is that it is the organizations that understand the value of having a healthy workforce, emphasizing the need to focus on measures that promote this goal.

A review from 2006 summarizes the international research linking healthy organizations to employee wellbeing. Links are identified and presented in five categories: work-life balance, employee growth and development, health and safety, recognition, and employee involvement. It also suggests that these links are contingent on the effectiveness of communication within the organization and the alignment of workplace practices with the organizational context. While these findings are relevant at the individual level, they also connect to the organizational health factors identified in the current study (19).

### 4.1 Methodological discussion and future research

In all organizational research, it is often difficult to control for other factors in the organizational context that might have an impact on the research question. This can make it difficult to attribute the results of a study solely to the particular phenomena being studied, in this case, the factors on an organizational level that have a health-promoting impact. This was addressed by interviewing several managers within the same organization and choosing a time when there were no major changes in the organizations, such as political elections.

The study uses sick-leave as a measure of health, which, while somewhat narrow, provides a concrete indication of whether the organization is able to have employees who stay healthy and can be active and productive in the organization. It would also be interesting to see if there is a difference between staff turnover as a measure of sustainability. Staff turnover is probably a measure that differs greatly depending on where in Sweden the organization is located and what the labor market looks like there.

This study's findings of factors on an organizational level behind low levels of sickness absence are theoretically related to well-known psychosocial and managerial factors related to the work environment. These work environment factors are essential for creating a healthy working life, which characterizes low sickness absence within organizations. These factors are considered stable over time and resilient to external societal pressures, including economic fluctuations.

Historically, these factors have been recognized as essential to workplace health. They address fundamental human needs for social support, personal development, and autonomy, as evidenced by Deci and Ryan's work on self-determination theory (70, 71). The classic demand-control-support model emphasizes the importance of participation and social support as stable, health-promoting elements in the workplace (72). Recent studies further affirm that management support is an important form of workplace support (32, 73–75). Another established model, Siegrist's effort-reward imbalance theory, highlights the significance of opportunities for development and constructive feedback, both vital for fostering workplace health (76). The findings also emphasize the importance of managerial style of the leaders and also their prerequisites related to the concept of organizational justice, which also can be seen as a stable factor (34). These foundational theories support the relevance of the factors identified in this study, regardless of external pressures.

More recent research on health-promoting workplaces has identified similar factors as those presented in the current study, reinforcing the validity of these findings (74, 77–79). The current research has garnered significant attention in Sweden, from trade unions, the health sector, occupational health services, and the Swedish Work Environment Authority (22, 23).

Given that these factors continue to hold importance, our results from the earlier Swedish report are likely to be of interest to a broader international audience. There appears to be a growing emphasis on measuring workplace factors rather than utilizing data to enhance worker health. While factors such as immigration and the COVID-19 pandemic certainly influence the work environment, the foundational concepts outlined remain vital.

Interview studies with a qualitative analysis method require methods to make generalization of the results feasible. This study ensured credibility through triangulation and respondent control. However, a limitation of interview studies is that interviewees may provide responses they think the researcher wants to hear, reflecting on what is socially desirable. To mitigate this, we conducted several interviews within the same organization.

Employee participation is recognized as a critical determinant of workplace health and wellbeing. Future research should explore the mechanisms through which change management strategies can be effectively implemented to enhance employee engagement. This could involve conducting intervention studies that examine the relationship between leadership transformations and corresponding shifts in employee behaviors, with a specific focus on how these changes influence participation levels. For instance, research could investigate how varying leadership styles or management approaches impact employee involvement and workplace communication. Understanding these links may provide valuable insights into optimizing organizational health and performance.

Future research should also focus on developing organizational health metrics that assess current conditions and predict potential work environment challenges, enabling continuous adjustments. These could include measures of employee engagement, perceived wellbeing, and productivity, while encouraging employee participation. Some initial work has been done in this area (80). Regular use of these tools, along with follow-up, will support continuous improvement, helping to refine assessments and improve the accuracy of outcome predictions.

## 5 Conclusions

The results indicate that municipalities with lower sickness absence rates tend to have more sophisticated organizational strategies, especially in clear and manageable areas. Creating a foundation for healthier organizations undoubtedly requires intricate strategic planning and well-defined structures, especially during periods of organizational change and development.

A recurring theme in the analysis is the proximity and interconnectedness observed in leadership, communication, and employee participation among the divisions, department managers, and employees of robust municipalities. This closeness is exemplified by leaders who actively participate in various operations, maintain regular communication, or have a comprehensive understanding of ongoing activities. Leaders in such organizations also often show a high degree of involvement in day-to-day activities. This phenomenon, known as the ‘proximity principle,' emerges as a potentially important factor influencing health outcomes, which should be explored further.

## Data Availability

The raw data supporting the conclusions of this article will be made available by the authors, without undue reservation.

## References

[1] Severin J Svensson M Akerstrom M. Cost-benefit evaluation of an organizational-level intervention program for decreasing sickness absence among public sector employees in Sweden. Int J Environ Res Public Health (2022) 19:2998. 10.3390/ijerph19052998 35270691 PMC8910127

[2] Stahl C Gustavsson IN Jonsdottir IH Akerstrom M. Multilevel, risk group-oriented strategies to decrease sickness absence in the public sector: evaluation of interventions in two regions in Sweden. Int Arch Occup Environ Health. (2022) 95:1415–27. 10.1007/s00420-022-01864-6 35451629 PMC9273540

[3] Montin S Granberg M. Moderna Kommuner. Stockholm: Liber (2021).

[4] Swedish Association of Local Authorities and Regions. Municipal Staff 2019 Stockholm, Sweden: Swedish Association of Local Authorities and Regions (2024). Available at: https://skr.se/skr/arbetsgivarekollektivavtal/uppfoljninganalys/personalstatistik/personalenisiffror/tabellerkommunalpersonal2023/tabellerkommunalpersonal2019.46930.html (accessed October 09, 2024).

[5] Dekkers-Sanchez PM Hoving JL Sluiter JK Frings-Dresen MH. Factors associated with long-term sick leave in sick-listed employees: a systematic review. Occup Environ Med. (2008) 65:153–7. 10.1136/oem.2007.034983 17881466

[6] Duijts SF Kant I Swaen GM van den Brandt PA Zeegers MP A. Meta-analysis of observational studies identifies predictors of sickness absence. J Clin Epidemiol. (2007) 60:1105–15. 10.1016/j.jclinepi.2007.04.008 17938051

[7] Head J Kivimaki M Martikainen P Vahtera J Ferrie JE Marmot MG. Influence of change in psychosocial work characteristics on sickness absence: the whitehall II study. J Epidemiol Community Health. (2006) 60:55–61. 10.1136/jech.2005.038752 16361455 PMC2465520

[8] Johansson G. The Illness Flexibility Model and Sickness Absence. Stockholm (2007).

[9] Marmot M Feeney A Shipley M North F Syme SL. Sickness absence as a measure of health status and functioning: from the UK whitehall II study. J Epidemiol Community Health. (1995) 49:124–30. 10.1136/jech.49.2.124 7798038 PMC1060095

[10] Melchior M Krieger N Kawachi I Berkman LF Niedhammer I Goldberg M. Work factors and occupational class disparities in sickness absence: findings from the gazel cohort study. Am J Public Health. (2005) 95:1206–12. 10.2105/AJPH.2004.048835 15933236 PMC1449341

[11] Niedhammer I Bugel I Goldberg M Leclerc A Gueguen A. Psychosocial factors at work and sickness absence in the gazel cohort: a prospective study. Occup Environ Med. (1998) 55:735–41. 10.1136/oem.55.11.735 9924449 PMC1757529

[12] Whitaker SC. The management of sickness absence. Occup Environ Med. (2001) 58:420–4. 10.1136/oem.58.6.420 11351060 PMC1740146

[13] Macdonald LA Harenstam A Warren ND Punnett L. Incorporating work organisation into occupational health research: an invitation for dialogue. Occup Environ Med. (2008) 65:1–3. 10.1136/oem.2007.033860 18089855

[14] Härenstam A. The Significance of Organisation for Healthy Work: Methods, Study Design, Analysing Strategies and Empirical Results from the Moa-Study. Stockholm: Arbetslivsinstitutet (2004).

[15] Härenstam A. Understanding the Organisational Impact on Working Conditions and Health. Stockholm: Arbetslivsinstitutet, förlagstjänst (2006).

[16] Marklund S Bolin M von Essen J. Can individual health differences be explained by workplace characteristics?–a multilevel analysis. Soc Sci Med. (2008) 66:650–62. 10.1016/j.socscimed.2007.09.008 17996347

[17] Biron C Burke RJ Cooper CL. Creating Healthy Workplaces: Stress Reduction, Improved Well-Being, and Organizational Effectiveness. Farnham: Gower (2014).

[18] Cameron KS Quinn RE. Diagnosing and Changing Organizational Culture: Based on the Competing Values Framework. San Francisco, Calif: Jossey-Bass (2011).

[19] Grawitch MJ Gottschalk M Munz DC. The path to a healthy workplace: a critical review linking healthy workplace practices, employee well-being, and organizational improvements. Consult Psychol J. (2006) 58:129–47. 10.1037/1065-9293.58.3.129

[20] Leka S Cox T Zwetsloot G. The European Framework for Psychosocial Risk Management. Nottingham, UK: Institute of Work, Health & Organisations, University of Nottingham (2008). p. 16.

[21] Eriksson T Christensen M. Positive Factors at Work: The First Report of the Nordic Project. Copenhagen: Nordic Council of Ministers (2008).

[22] Swedish Agency for Work Environment Expertise (SAWEE) Swedish Work Environment Authority. Friskfaktorer Som Kan Mätas Och Följas Över Tid [Elektronisk Resurs]: Swedish Agency for Work Environment Expertise (SAWEE), Swedish Work Environment Authority (2021).

[23] Hultberg A Ingibjörg Hrönn J Ahlborg G Winroth J Corin L Heimdahl M. Hälsa På Arbetsplatsen: En Sammanställning Av Kunskap Och Metoder. [Göteborg]: Institutet för stressmedicin (2018).

[24] Cameron KS Dutton JE Quinn RE. Positive Organizational Scholarship: Foundations of a New Discipline. San Francisco, CA: Berrett-Koehler (2003).

[25] Cameron KS Spreitzer GM. The Oxford Handbook of Positive Organizational Scholarship. Oxford: Oxford University Press (2013).

[26] Luthans F Youssef CM. Emerging positive organizational behavior. J Manage. (2007) 33:321–49. 10.1177/0149206307300814

[27] Bakker AB Derks D. “Positive occupational health psychology.” *Occupational Health Psychology*. Hoboken, NJ, US: Wiley Blackwell (2010). p. 194–224.

[28] Baxter R Taylor N Kellar I Lawton R. What methods are used to apply positive deviance within healthcare organisations? a systematic review. BMJ Qual Saf. (2016) 25:190–201. 10.1136/bmjqs-2015-004386 26590198 PMC4789698

[29] Pfeffer J. Building sustainable organizations: the human factor. Acad Manag Perspect. (2010) 24:34–45. 10.5465/AMP.2010.50304415

[30] Pfeffer J. Producing sustainable competitive advantage through the effective management of people. Acad Manag Perspect. (1995) 9:55–69. 10.5465/ame.1995.9503133495

[31] Pfeffer J. Seven practices of successful organizations. Calif Manage Rev. (1998) 40:96–124. 10.2307/41165935

[32] Kuoppala J Lamminpää A Liira J Vainio H. Leadership, job well-being, and health effects–a systematic review and a meta-analysis. J Occup Environ Med. (2008) 50:904–15. 10.1097/JOM.0b013e31817e918d 18695449

[33] Crewe S Girardi A. Nurse managers: being deviant to make a difference. J Manag Organ. (2020) 26:324–39. 10.1017/jmo.2019.72

[34] Stoetzer U Aborg C Johansson G Svartengren M. Organization, relational justice and absenteeism. Work. (2014) 47:521–9. 10.3233/WOR-131624 23531587

[35] Stoetzer U Bergman P Aborg C Johansson G Ahlberg G Parmsund M. Organizational factors related to low levels of sickness absence in a representative set of swedish companies. Work. (2014) 47:193–205. 10.3233/WOR-2012-1472 22976159

[36] Svartengren M Stoetzer U Parmsund M Eriksson T Stöllman Å Vingård E. Report 1/2013. Hälsa Och Framtid I Kommuner Och Landsting Uppsala, Sweden (2013).

[37] Thornblad H Lorentzen D. Nycklar Till Friska Företag: Inspirerande Exempel Och Resultat Från Forskningsprojektet Hälsa Och Framtid: Prevent (2016).

[38] Brinkmann S Kvale S. Interviews: Learning the Craft of Qualitative Research Interviewing. Los Angeles: Sage Publications (2015).

[39] Tong A Sainsbury P Craig J. Consolidated criteria for reporting qualitative research (Coreq): a 32-item checklist for interviews and focus groups. Int J Qual Health Care. (2007) 19:349–57. 10.1093/intqhc/mzm042 17872937

[40] Graneheim UH Lindgren BM Lundman B. Methodological challenges in qualitative content analysis: a discussion paper. Nurse Educ Today. (2017) 56:29–34. 10.1016/j.nedt.2017.06.002 28651100

[41] Graneheim UH Lundman B. Qualitative content analysis in nursing research: concepts, procedures and measures to achieve trustworthiness. Nurse Educ Today. (2004) 24:105–12. 10.1016/j.nedt.2003.10.001 14769454

[42] Hsieh HF Shannon SE. Three approaches to qualitative content analysis. Qual Health Res. (2005) 15:1277–88. 10.1177/1049732305276687 16204405

[43] QSR International. Nvivo 9. 9 ed. Burlington, Massachusetts, USA (2010). Available at: https://www.qsrinternational.com/nvivo-qualitative-data-analysis-software/home

[44] Bjorklund C Lohela-Karlsson M Jensen I Bergstrom G. Hierarchies of health: health and work-related stress of managers in municipalities and county councils in Sweden. J Occup Environ Med. (2013) 55:752–60. 10.1097/JOM.0b013e318295681c 23787564

[45] van der Doef M Maes S Diekstra R. An Examination of the job demand-control-support model with various occupational strain indicators. Anx Stress Coping. (2000) 13:165–85. 10.1080/1061580000824833835955099

[46] Tafvelin S Lundmark R von Thiele Schwarz U Stenling A. Why do leaders engage in destructive behaviours? The role of leaders' working environment and stress journal of occupational and organizational. Psychology. (2023) 96:165–81. 10.1111/joop.12413

[47] Statistics Sweden (SCB). Share of Sick Leave in Different Sectors [Table]: Statistics Sweden (SCB) (2024). Available at: https://www.statistikdatabasen.scb.se (accessed September 29, 2024).

[48] Colquitt JA Conlon DE Wesson MJ Porter COLH Ng KY. Justice at the millennium: a meta-analytic review of 25 years of organizational justice research. J Appl Psychol. (2001) 86:425–45. 10.1037/0021-9010.86.3.425 11419803

[49] Colquitt JA Scott BA Rodell JB Long DM Zapata CP Conlon DE. Justice at the millennium, a decade later: a meta-analytic test of social exchange and affect-based perspectives. J Appl Psychol. (2013) 98:199–236. 10.1037/a0031757 23458336

[50] Elovainio M Ferrie JE Gimeno D De Vogli R Shipley M Brunner EJ. Organizational justice and sleeping problems: the whitehall II study. Psychosom Med. (2009) 71:334–40. 10.1097/PSY.0b013e3181960665 19251877

[51] Elovainio M Kivimaki M Vahtera J. Organizational justice: evidence of a new psychosocial predictor of health. Am J Public Health. (2002) 92:105–8. 10.2105/AJPH.92.1.105 11772771 PMC1447369

[52] Ferrie JE Head J Shipley MJ Vahtera J Marmot MG Kivimaki M. Injustice at work and incidence of psychiatric morbidity: the whitehall II study. Occup Environ Med. (2006) 63:443–50. 10.1136/oem.2005.022269 16698805 PMC2092506

[53] Head J Kivimaki M Siegrist J Ferrie JE Vahtera J Shipley MJ. Effort-reward imbalance and relational injustice at work predict sickness absence: the whitehall II study. J Psychosom Res. (2007) 63:433–40. 10.1016/j.jpsychores.2007.06.021 17905053

[54] Moliner C Martínez-Tur V Peiró JM Ramos J Cropanzano R. Relationships between organizational justice and burnout at the work-unit level. Int J Stress Manag. (2005) 12:99–116. 10.1037/1072-5245.12.2.99

[55] Spell CS Arnold TJ A. Multi-level analysis of organizational justice climate, structure, and employee mental health. J Manage. (2007) 33:724–51. 10.1177/0149206307305560

[56] Eriksson A. Health-Promoting Leadership: A Study of the Concept and Critical Conditions for Implementation and Evaluation. Nordic School of Public Health (2011).

[57] Nyberg A. “Det Goda Chefskapet. Organisatorisk Effektivitet Och Anställdas Hälsa En Kunskapsöversikt.” In: Döös M Waldenström K, editors. Chefskapets Former Och Resultat Stockholm. Sweden: VINNOVA (2008).

[58] Nyberg A. The Impact of Managerial Leadership on Stress and Health among Employees. Stockholm: Karolinska Institutet (2009).

[59] de Jonge J Kompier MAJ. A critical examination of the demand-control-support model from a work psychological perspective. Int J Stress Manag. (1997) 4:235–58. 10.1023/B:IJSM.0000008152.85798.90

[60] Johnson JV Hall EM. Job Strain, work place social support, and cardiovascular disease: a cross-sectional study of a random sample of the swedish working population. Am J Public Health. (1988) 78:1336–42. 10.2105/AJPH.78.10.1336 3421392 PMC1349434

[61] Karasek RA Jr. Job demands, job decision latitude, and mental strain: implications for job redesign. Adm Sci Q. (1979) 24:285–308. 10.2307/2392498

[62] Karasek R Brisson C Kawakami N Houtman I Bongers P Amick B. The job content questionnaire (JCQ): an instrument for internationally comparative assessments of psychosocial job characteristics. J Occup Health Psychol. (1998) 3:322–55. 10.1037//1076-8998.3.4.322 9805280

[63] Theorell T De Manzano O Lennartsson AK Pedersen NL Ullen F. Self-reported psychological demands, skill discretion and decision authority at work: a twin study. Scand J Public Health. (2016) 44:354–60. 10.1177/1403494815626610 26825630

[64] Theorell T Hammarstrom A Aronsson G Traskman Bendz L Grape T Hogstedt C. A systematic review including meta-analysis of work environment and depressive symptoms. BMC Public Health. (2015) 15:738. 10.1186/s12889-015-1954-4 26232123 PMC4522058

[65] Arnold KA. Transformational leadership and employee psychological well-being: a review and directions for future research. J Occup Health Psychol. (2017) 22:381–93. 10.1037/ocp0000062 28150998

[66] Arnold KA Turner N Barling J Kelloway EK McKee MC. Transformational leadership and psychological well-being: the mediating role of meaningful work. J Occup Health Psychol. (2007) 12:193–203. 10.1037/1076-8998.12.3.193 17638487

[67] Bass BM. Two decades of research and development in transformational leadership. Eur J Work Organ Psychol. (1999) 8:9–32. 10.1080/135943299398410

[68] Provision Afs 2001:1. Systematiskt Arbetsmiljöarbete (2001).

[69] Provision Afs 2015:4. Organisational and Social Work Environment (2015).

[70] Deci EL Ryan RM. Intrinsic Motivation and Self-Determination in Human Behavior. New York: Springer Science+Business Media (1985).

[71] Deci EL Ryan RM. The “what” and “why” of goal pursuits: human needs and the self-determination of behavior. Psychol Inq. (2000) 11:227–68. 10.1207/S15327965PLI1104_01

[72] Karasek R Theorell T. Healthy Work: Stress, Productivity, and the Reconstruction of Working Life. New York, NY: Basic Books (1990).

[73] Jiménez P Bregenzer A Kallus KW Fruhwirth B Wagner-Hartl V. Enhancing resources at the workplace with health-promoting leadership. Int J Environ Res Public Health. (2017) 14:1264. 10.3390/ijerph14101264 29053640 PMC5664765

[74] Mullen J Thibault T Kelloway EK. “Occupational Health and Safety Leadership.” *Handbook of Occupational Health Psychology, 3rd Ed*. Washington, DC, US: American Psychological Association (2024). p. 501–16. 10.1037/0000331-0025

[75] Vidman Å Strömberg A. Leadership for a healthy work environment–a question about who, what and how. Lead Health Serv. (2020) 34:1–15. 10.1108/LHS-06-2020-0041 33818972 PMC8297597

[76] Siegrist J. “Effort-reward imbalance at work and health.” *Historical and Current Perspectives on Stress and Health*. Research in Occupational Stress and Well-Being. US: Elsevier Science/JAI Press (2002). p. 261–91.

[77] Nielsen K. Organizational Interventions. Oxford: Oxford University Press (2022).

[78] Shiri R El-Metwally A Sallinen M Pöyry M Härmä M Toppinen-Tanner S. The role of continuing professional training or development in maintaining current employment: a systematic review. Healthcare. (2023) 11:2900. 10.3390/healthcare11212900 37958044 PMC10647344

[79] Öhrling T. Increased participation among cleaners as a strategy to improve quality and occupational health. Nord J Work Life Stud. (2014) 4:79–98. 10.19154/njwls.v4i3.418139659712

[80] Molin F Paulsson S Hellman T Svartengren M. Can the human resources index (HRI) be used as a process feedback measurement in a structured support model for systematic work environment management? Int J Environ Res Public Health. (2021) 18:6509. 10.3390/ijerph18126509 34208784 PMC8296489

